# Development of Priority Outcome Domains for Community Mental Health Research via Consensus Among Multiple Stakeholders: Online Delphi Study in Japan

**DOI:** 10.1111/inm.70049

**Published:** 2025-05-14

**Authors:** Takuma Shiozawa, Sosei Yamaguchi, Momoka Igarashi, Makoto Ogawa, Makiko Abe, Naonori Yasuma, Takayuki Kawaguchi, Chiyo Fujii

**Affiliations:** ^1^ Department of Mental Health and Psychiatric Nursing Graduate School of Health Care Sciences, Institute of Science Tokyo Tokyo Japan; ^2^ Department of Community Mental Health & Law National Institute of Mental Health, National Center of Neurology and Psychiatry Tokyo Japan; ^3^ Ageonomori Clinic Saitama Japan

**Keywords:** community mental health services, Delphi method, outcome assessments, patient participation, stakeholder participation

## Abstract

Several core outcome sets (COS) have been developed in the field of mental health; however, the specific outcome domains that should be prioritised in community mental health research remain unclear. This study aimed to identify the key outcome domains for community mental health research in Japan, as determined by multiple stakeholders. First, a comprehensive list of outcome domains was compiled by scoping reviews, focus group interviews, and web‐based questionnaire surveys. This was followed by two rounds of preliminary surveys with multiple stakeholders. Finally, three rounds of a web‐based Delphi survey were conducted to determine the most important outcome domains of community mental health research in Japan. A total of 96 outcome domains were evaluated by 297 participants, with a response rate of 93.6%. This study identified 24 outcome domains that are essential for community mental health research in Japan. These 24 domains, which reached a consensus, included aspects related to comprehensive symptom assessment and personal recovery. Given that community care serves patients with diverse illnesses, identifying outcome domains that are broadly applicable across different community care settings rather than being limited to specific conditions is critical for future research. The study also highlights differences in perspectives among stakeholders regarding the importance of various outcomes. Despite these differences, several outcome domains have been recognised as being significantly important in the overall consensus.

**Trial Registration:** University Hospital Medical Information Network (UMIN) Clinical Trials Registry: UMIN000044680.

## 1 Background

Inadequate outcome selection negatively impacts both appropriate care provision and meaningful research. Without standardised outcome criteria, inconsistencies in reporting results emerge, complicating the comparison of findings across studies. This issue impedes the progress of evidence‐based practices and undermines the precision and relevance of community mental health studies. In Japan, deinstitutionalisation of patients with mental disorders has led to more community‐dwelling service users and a transformation of the service delivery system. With the increase in the number of community‐dwelling patients with mental illness, researchers have gradually focused on outcomes related to social and subjective factors (e.g., employment and interpersonal interactions) in addition to clinical outcomes (e.g., readmission, symptoms, and functioning) (Thornicroft and Slade 2014). Personal recovery, a concept developed by user movements, emphasises outcomes aligned with the patient's own goals and values, distinct from clinical recovery (Slade et al. 2008). In this context, patient‐reported measures have gained increasing importance as key outcomes in clinical research (Yamaguchi, Ojio, et al. 2024; Yamaguchi, Usui, et al. 2024). Despite the diversification of outcomes to enhance community care, the selection of appropriate outcomes remains a concern.

To support evidence‐based practice, initiatives such as the development of the core outcome set (COS) are underway to clarify the criteria for selecting outcomes (COMET 2011). A COS is a standardised set of outcomes essential for all clinical trials within a health or medicine domain, facilitating the integration of study results and enhancing evidence value. COS development follows a stepwise approach, starting with the identification of core outcome domains and setting measurement standards for each domain (COMET 2011). In addition, during COS development, reaching a consensus among various stakeholders is necessary to enhance the reliability of outcome selection (Dodd et al. 2023). For instance, Zendjidjian and Boyer (2014) recommended the involvement of patients' families and caregivers in defining outcomes and designing questionnaires related to deinstitutionalization and community mental health development. Collins (2019) highlighted differing perspectives among individuals with mental disorders and professional support staff regarding defining outcomes. Recent global trends suggest that incorporating the concept of patient and public involvement (PPI), along with the perspectives of service users and other stakeholders, is crucial in mental health research (Boardman 2018). Furthermore, PPI is recognised as a valuable approach not only in intervention studies but also in outcome selection research (Smith et al. 2018). Indeed, although numerous clinical studies have focused on assessing symptom severity, a Japanese qualitative study has indicated that individuals with schizophrenia tend to prefer outcome scales containing their personal values and spirituality. (Sawada et al. 2024). A COS should be an internationally applicable, standardised set of outcomes to be measured and reported. Meanwhile, because important issues in personal recovery and individuals' lives are influenced by culture and beliefs (Leamy et al. 2011), research in each cultural sphere is also important when examining psychosocial outcomes.

The need for COS in community mental health is evident, with specific ones developed for various diagnoses and hospital settings such as depression and anxiety disorders (Obbarius et al. 2017), psychotic disorders (Mckenzie et al. 2022), and bipolar disorder in community‐dwelling patients (Retzer et al. 2020). A COS for evaluating interventions in hospitalised patients has been developed (Tyler et al. 2020). In summary, COSs that have been developed to date primarily focus on specific diagnoses and instances of hospitalisation. In other words, there is a lack of standard outcome domains that are crucial for mental health service users living in the community. In particular, COS for specific diagnoses might not be able to fit into a community care context, which extends beyond diagnosis, since community service providers usually treat people with a wide variety of psychiatric diagnoses and living issues. Therefore, COS development for mental health in the community context with a specific cultural background is needed to effectively evaluate community care systems.

This study aimed to explore the consensus on important outcome domains through Delphi research among multiple stakeholders in community mental health care and research in Japan. The novelty of this study lies in its identification of crucial outcome domains within community mental health without focusing on specific diagnoses. The target population within the framework of community mental health includes individuals with various diagnoses. Ensuring diverse service user participation, including patient participation, is critical for disseminating actionable research findings (Obbarius et al. 2017; Mckenzie et al. 2022) and addressing patient‐relevant concerns (Kelly et al. 2015). Stakeholders in this context include service users, caregivers, service providers, government officials, and researchers who can influence community mental health research. This study encourages collaboration to capture a wide range of perspectives and needs effectively.

## 2 Methods

### 2.1 Delphi Process

In this study, three rounds of the online Delphi method were conducted from August 25, 2021, to November 14, 2021. Participants were stakeholders in community mental health research: service users, caregivers, service providers, government officials, and researchers. The Delphi method is used to obtain consensus from multiple stakeholders. This method was developed by the RAND Corporation in the 1950s and defined as ‘a set of procedures for condensing and refining the opinions of a group of people (Dalkey 1969)’. The flexibility of the Delphi method makes it suitable for understanding phenomena, objectives, and problems and for consolidating opinions from diverse stakeholders. In effect, a recent review involving COS development reports that the use of the Delphi technique for assessing and developing consensus has risen from 15% to 31%, indicating that it is an approach that has been gaining attention (Gorst et al. 2016). Therefore, we have chosen this method for our study. The Delphi method is widely recognised as an effective approach for consolidating the opinions of patients, families, and a diverse range of stakeholders who support patients, beyond its application in the development of COS (Jairath and Weinstein 1994). Although the Delphi method does not facilitate direct interaction among participants, it allows a substantial number of participants to contribute independently and anonymously. This method is particularly advantageous in engaging many geographically dispersed participants (Sinha et al. 2011). A notable benefit of the Delphi method is that it does not necessitate participants to immediately present their opinions collectively or to articulate their views in front of a group. Instead, it provides participants the flexibility to respond at their own pace, within a given timeframe. This characteristic makes the Delphi method particularly well suited for facilitating the participation of a diverse array of stakeholders, such as patients with mental illnesses and their families, and for aggregating their opinions.

**FIGURE 1:**
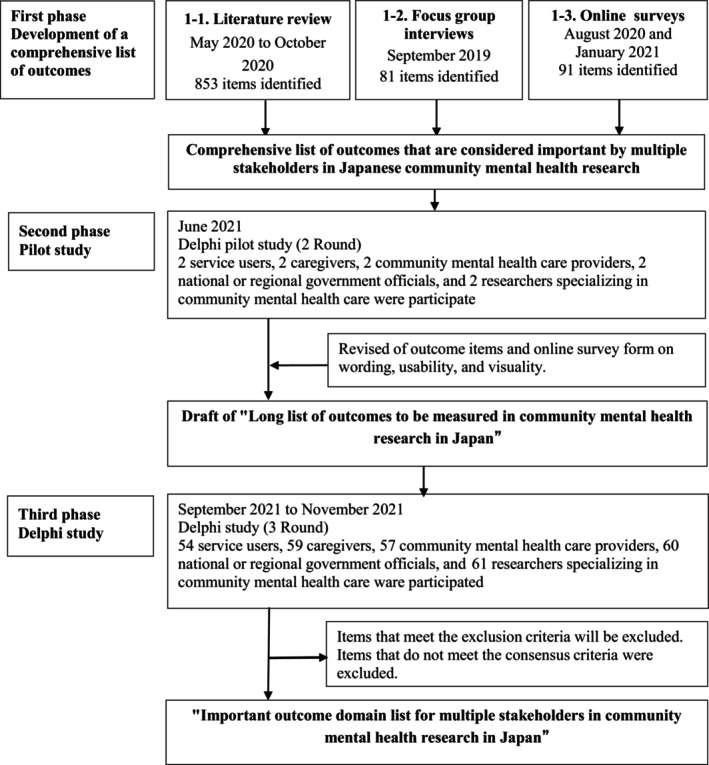
Research process.

We followed a three‐phase approach to conduct the Delphi survey. In the first phase, we integrated past research findings and current stakeholder perspectives on community mental health to create a comprehensive list of outcomes. Unlike classical Delphi methods, which often begin with the generation of items from open‐ended questions, items were gathered through a combination of literature review, group interviews, and online questionnaire surveys for this study. This methodology allowed for broader and more inclusive collection of data, ensuring that the outcomes reflected both historical research and contemporary practices in community mental health. Some results from each survey have already been published (Igarashi et al. 2021; Yasuma et al. 2022). Outcomes with commonalities or overlapping elements were grouped together to form outcome domains. After several meetings among research team members, we established 94 outcome domains and created a summary to understand the meaning of each item. In the second phase, we refined the long list of outcome domains in a pilot study on the basis of preliminary survey results, resulting in 96 outcome domains for the Delphi survey (Table S1). In the third phase, we conducted a three‐round Delphi survey using the long list of the outcome domains (Figure 1). The research team consisted of researchers with expertise in medicine or welfare and researchers with service user experience (user researchers). The protocol paper provides a detailed overview of the survey methods used in this study (Shiozawa et al. 2021). We adhered to the Guidance on Conducting and REporting DElphi Studies where applicable (Junger et al. 2017).

### 2.2 Participants and Recruitment

Individuals eligible for participation in the Delphi survey included stakeholders in community mental health care: service users, caregivers, community mental health service providers, government officials, and researchers. We expected 50 participants from each stakeholder group; on the basis of an 11.7% attrition rate from a previous study (Fukasawa et al. 2015), we recruited approximately 60 participants from each stakeholder group. The inclusion criteria were (1) ability to voluntarily participate in the study and (2) residency in Japan. Exclusion criteria were (1) current hospitalisation, (2) age under 20 years, and (3) adult guardianship. Details regarding panel selection can be found in the protocol paper (Shiozawa et al. 2021).

We approached various organisations relevant to community mental health, including groups representing service users, caregivers, professionals, government officials, and academic communities, to explain our study's objectives and content. These organisations were selected based on their active involvement in supporting stakeholders and their relevance to community mental health, ensuring diverse viewpoints in our participant pool. After obtaining cooperation from key individuals within these organisations, they assisted in disseminating study information via organisational mailing lists and email newsletters. Furthermore, individuals who had participated in previous related studies were contacted, along with authors of pertinent original articles focusing on community psychiatry and mental health services in Japan. During the recruitment process, we provided a thorough explanation of the study details and burden associated with participation before requesting voluntary participation. Participants were informed upon recruitment that they would receive 500 yen for each round of participation.

### 2.3 Delphi Rounds/Data Collection

We conducted three rounds of Delphi surveys, where participants evaluated the 96 items on a seven‐point scale, ranging from ‘very important (7)’ to ‘not important at all (1)’. In addition, participants could provide comments if they had opinions on each item. Items that received ‘very important (7)’ and ‘important (6)’ responses from over 70% of participants were considered to have reached consensus and were excluded from subsequent rounds. Similarly, items that received ‘not important at all (1)’ and ‘not important (2)’ responses from more than 75% of the participants were considered to have not reached consensus and were excluded. The remaining items for which consensus was not achieved were presented again for further evaluation. The open‐ended responses for each item were provided as feedback to all participants along with analysis results, such as the median and interquartile range (IQR) of the importance score for each item. Owing to the high volume of free‐text responses, the research team also created a summary to facilitate participants' understanding (Table S2). Details about the Delphi survey, such as the consensus and non‐consensus criteria and the number of points on the scale, were outlined in the protocol paper (Shiozawa et al. 2021). To maintain data accuracy, we did not replace participants who dropped out, but we took measures to minimise the dropout rate. By providing participants with extensive information about the study in advance, as suggested by Avella (2016), and sending reminder emails and offering points of contact for direct communication with researchers, we aimed to create an environment that encouraged participation.

### 2.4 Analysis

For each item, we calculated the importance score of all participants and compared scores among the different stakeholder groups. First, we computed the median and IQR for each stakeholder group. Second, we conducted a Kruskal–Wallis test followed by Dunn's post hoc test. The significance level was 5% for multiple comparisons. All analyses were performed using Stata version 16.

### 2.5 Patient and Public Involvement in This Research

This study incorporated the views of stakeholders at various stages of the research process. Specifically, the protocol development phase was conducted in consultation with researchers who have lived experience as service users (user researchers). Individuals with mental illnesses and user researchers provided input during the creation of a long list of outcomes and survey websites. In the second phase, a diverse group of stakeholders was recruited as research collaborators to provide feedback on survey procedures and the long list of outcomes created. This feedback was incorporated in an iterative process that gathered further opinions, reflecting a wider range of stakeholder perspectives.

### 2.6 Ethical Approval and Informed Consent

The study protocol (Shiozawa et al. 2021) was approved by the Ethics Committee of Tokyo Metropolitan University (Approval No. 20083) and the Research Ethics Committee of the National Center of Neurology and Psychiatry (Approval No. A2021‐005). The protocol was registered with the University Hospital Medical Information Network (UMIN) Clinical Trials Registry. All participants provided informed consent online in Japanese via the survey platform, after confirming that they had understood the study procedures and requirements.

## 3 Results

### 3.1 Participants

During the recruitment process, 421 individuals expressed an interest in participating. After stratifying by stakeholder group, random selection was conducted, resulting in 302 participants being chosen. Table 1 provides detailed information on the participants who responded to the first round survey (*n* = 291).

**TABLE 1:** Characteristic of participants to Delphi survey.

		Service user, *n* = 54		Caregiver, *n* = 59		Service provider, *n* = 57		Government official, *n* = 60		Researcher, *n* = 61	
		(Ave/*N*)	(SD/%)	(Ave/*N*)	(SD/%)	(Ave/*N*)	(SD/%)	(Ave/*N*)	(SD/%)	(Ave/*N*)	(SD/%)
Age		43.6	9.4	57.1	11.0	44.1	11.0	44.5	8.0	46	8.7
Sex	Male	27	50.0	21	35.6	31	54.4	34	56.7	26	42.6
	Female	26	48.2	38	64.4	26	45.6	26	43.3	35	57.4
	Others	1	1.9	0	0.0	0	0.0	0	0.0	0	0.0
Years of experience*		19.5	9.1	14.5	10.9	14.5	9.0	13.8	9.6	10.8	7.2
Diagnosis**	Schizophrenia	18	33.3	44	74.6	—	—	—	—	—	—
	Bipolar disorder	13	24.1	6	10.2	—	—	—	—	—	—
	Depression	9	16.7	1	1.7	—	—	—	—	—	—
	Developmantal disorder	5	9.3	1	1.7	—	—	—	—	—	—
	Anxiety disorder (panic disorder, obsessive‐compulsive disorder)	1	1.9	0	0.0	—	—	—	—	—	—
	Others	8	14.8	6	10.2	—	—	—	—	—	—
Relation	Parents	—	—	33	55.9	—	—	—	—	—	—
	Sibling	—	—	11	18.6	—	—	—	—	—	—
	Spouse	—	—	11	18.6	—	—	—	—	—	—
	Child	—	—	2	3.4	—	—	—	—	—	—
	Others	—	—	2	3.4	—	—	—	—	—	—
Certification	Doctor	—	—	—	—	10	17.5	—	—	6	9.8
	Nurse	—	—	—	—	10	17.5	—	—	26	42.6
	Occupational therapist	—	—	—	—	10	17.5	—	—	9	14.8
	Social worker	—	—	—	—	21	36.8	—	—	8	13.1
	Clinical psychologist	—	—	—	—	6	10.5	—	—	9	14.8
	Others	—	—	—	—	0	0.0	—	—	1	1.6
	None	—	—	—	—	0	0.0	—	—	2	3.3
Academic history	Degree	—	—	—	—	—	—	—	—	4	6.6
	Master	—	—	—	—	—	—	—	—	19	31.2
	Doctor	—	—	—	—	—	—	—	—	38	62.3

* Service user: disease duration, caregiver: care duration, service provider and government staff: provision of community mental health or welfare service, Researcher: years of research activity.

** Caregiver: family's diagnosis.

### 3.2 Summary of Rounds

Table 2 summarises the responses during each round, including the number of respondents (response rate), items that reached consensus, and items that did not reach consensus. In the first round, 297 participants consented to participate in the survey, of which 291 submitted responses (response rate 98.0%). In the first round, 15 items reached consensus; 81 items that did not reach consensus were re‐evaluated in the next round. In the second round, 291 participants were invited to participate in the survey; 286 participants submitted responses (response rate 98.3%). In the second round, consensus was reached for 5 items; 76 items did not reach consensus and remained in the final round. In the third round, 286 participants were invited to participate in the survey, of which 278 participants submitted responses (response rate 97.2%). In the third round, 4 items reached consensus, and 72 items did not reach consensus.

**TABLE 2:** Summary of responses for each round.

	Number of respondents	Response rate (%)	Number of outcome domains	Outcome domains that reached consensus	Outcome domains that not consensus and re‐evaluated the next round	Outcome domains that removed or rejected
Round 1	291	98.0	96	15	81	0
Round 2	286	98.3	81	5	76	0
Round 3	278	97.2	76	4	72	0
Total	—	93.6	—	24	—	0

After three rounds of the Delphi survey, 24 of 96 items reached consensus (Table 3). Among the outcome domains established for caregivers, two items, family mental health and family emotional expression, met the consensus criteria. Of 297 participants who consented to the survey, 278 participated in all three rounds, resulting in a final response rate of 93.6% across all surveys (Tables 2 and 3).

**TABLE 3:** Percentage of agreement on outcome domains across the online Delphi survey.

		Round 1	Round 2	Round 3
Number of respondents (*N*)		291	286	278
Response rate (%)		98.0	98.3	97.2
Number	Items	Percentage agreement (%)	Percentage agreement (%)	Percentage agreement (%)
24	Help‐seeking	**81.1**	—	—
30	Place of safety and belonging	**81.1**	—	—
8	Suicidal ideation or attempt	**79.4**	—	—
42	Life satisfaction, quality of life, or well‐being	**77.7**	—	—
48	Sense of control and coping with symptoms	**72.9**	—	—
78	Adverse events or side effects	**72.5**	—	—
79	Caregivers' mental health	**72.5**	—	—
28	Social connectedness	**71.8**	—	—
55	Treatment adherence	**71.8**	—	—
44	Empowerment or self‐determination	**71.5**	—	—
58	Sufficiency level of needs or Unmet needs	**71.5**	—	—
66	Relationship with supporters	**71.5**	—	—
56	Attitudes toward medication or treatment	**71.1**	—	—
63	Family relationships or functioning	**71.1**	—	—
88	Expressed emotions	**70.1**	—	—
20	Daily living skills	67.4	**72.7**	—
60	Housing stability	67.7	**71.7**	—
45	Self‐esteem	68.7	**71.0**	—
17	Stress	68.4	**71.0**	—
1	Psychiatric symptoms or mental state	69.1	**70.3**	—
7	Violence or aggression	66.7	67.8	**72.0**
54	Medication adherence	64.9	67.5	**72.0**
9	Death—suicide	65.3	67.8	**71.6**
3	Relapse or remission	65.6	63.6	**70.1**
77	Medication prescription	68.4	67.1	68.0
85	Caregivers' knowledge of illness and services	66.7	68.5	68.0
92	Burden of care	68.4	67.8	68.0
57	Satisfaction with services	67.7	62.6	67.3
90	Social support for caregivers	69.4	68.2	67.3
93	Financial burden of care	64.3	64.3	67.3
94	Influence on caregivers' lifestyles	65.6	68.2	66.9
95	Influence on caregivers' jobs	62.2	64.7	66.9
53	Knowledge of illness and services	61.5	63.3	66.5
83	Caregivers' problem‐solving or coping skills	68.4	69.6	66.5
2	Anxiety and depression	68.0	64.7	65.8
22	Interpersonal relations	65.3	60.1	65.8
43	Motivation	62.9	62.2	64.8
12	Physical health	66.3	54.5	63.3
82	Family's stigma and discrimination	60.8	60.8	62.6
19	Overall functioning	61.5	61.9	61.5
52	Cognitive functioning	59.5	55.2	61.5
59	Perceived coercion	63.9	65.4	61.5
91	Experience of caregiving	59.5	61.5	61.5
26	Overall social functioning	58.8	64.3	61.2
27	Independent living	55.0	55.6	61.2
6	Self‐harm	55.3	58.7	60.8
49	Stigma and discrimination	58.1	61.2	60.8
4	Clinical insight	60.8	58.7	60.4
72	Involuntary treatment	57.0	59.8	59.7
71	Involuntary treatment hospitalisation	53.6	54.9	59.4
87	Caregivers' life satisfaction or quality of life	58.8	56.6	59.0
33	Desired nature and form of employment	50.9	53.5	58.6
5	Substance use	52.6	50.3	57.6
46	Resilience	52.2	55.6	57.6
80	Caregivers' physical health	62.2	57.0	57.2
84	Caregivers' self‐esteem	54.6	56.6	56.8
23	Communication skills and empathy	52.9	53.8	56.1
96	Influence on caregivers' leisure activities	54.6	54.9	56.1
10	Death—all causes	50.9	55.2	55.4
18	Subjective health status	56.4	61.5	55.0
65	Having a role model	55.7	51.0	54.7
89	Caregivers' service use	51.9	52.8	52.9
32	Employment status (all forms of employment)	40.5	48.3	52.2
86	Caregivers' service satisfaction	55.7	53.8	52.2
39	Education	48.1	43.4	51.8
61	Earnings	54.0	57.7	51.8
29	Activities or leisure	49.5	44.8	51.4
40	Role in society	54.6	52.4	51.4
11	Laboratory measures	51.5	46.5	51.1
15	Chronic pain	47.4	44.1	49.6
16	Self‐care	54.3	49.3	48.9
69	Mental health service use	46.0	47.2	48.9
81	Caregivers' subjective health status	49.1	50.3	48.9
62	Duration of community living	45.7	48.6	48.6
41	Peer support	46.4	45.5	47.5
68	Costs of all care	45.4	43.7	46.4
73	Outpatient visits	38.1	42.7	45.7
74	Emergency service use	38.8	43.0	43.9
37	Childbirth/child care	47.1	43.7	43.5
34	Work‐related skills or vocational ability	34.4	41.6	43.2
67	Costs of mental health care	44.0	41.3	41.7
76	Number of caregivers needed to maintain stable state	43.0	42.7	41.7
21	Contact with the legal system	34.7	37.4	41.4
47	Feeling dependent on psychiatric treatments	41.9	38.5	41.0
70	Hospital admission	35.1	38.5	41.0
75	Non‐psychiatric service use	38.8	38.1	39.9
35	Duration of employment, Absence from or leaving a job	36.8	40.2	39.3
64	Living with family	37.4	38.8	36.0
36	Job hunting and related activities	30.2	31.1	35.6
31	Employment status (general employment only)	25.4	24.8	33.8
38	Caregiving for family members	42.3	33.9	33.1
14	Physical fitness	30.3	29.0	30.6
13	Weight and obesity	28.5	27.6	29.5
25	Partner or marriage	29.2	23.8	28.4
51	Sexual satisfaction	25.4	25.5	27.0
50	Religion/beliefs	25.8	22.4	20.1

*Note:* Bold values indicate the items on which consensus was reached.

### 3.3 Comparison Between Stakeholder Groups

In each Delphi round, we conducted the Kruskal–Wallis test and Dunn's post hoc test for multiple comparisons to analyse score differences between stakeholder groups. We observed significant differences between stakeholder groups in 81 of 96 items (84.4%) in the first round, 72 of 81 items (88.9%) in the second round, and 60 of 76 items (78.9%) in the third round (Table S3).

## 4 Discussion

This study examines high‐priority outcome domains for Japanese community mental health research among multiple stakeholder groups. The three‐round Delphi survey identified 24 outcome domains that reached consensus as being important in community mental health research. The outcome domains that reached consensus in this Delphi survey were anticipated to have high affinity and relevance for stakeholders.

### 4.1 Outcome Domains Similar to Those in Previous Studies

In this study, consensus was reached on 24 outcome domains. Several of them were aligned with those of previous research: life satisfaction, quality of life, or well‐being; sense of control and coping with symptoms; adverse events or side effects; social connectedness; empowerment or self‐determination; relationship with supporters; attitude toward medication or treatment; family relationships or functioning; daily living skills; housing stability; self‐esteem; treatment adherence; and relapse or remission. Synonymous content was identified as core outcomes in a prior study on bipolar disorder (Retzer et al. 2020).

In a Delphi surveys on recovery of patients with mental illness, factors such as housing stability, support from others, positive relationships with others, empowerment, agency, and leading a fulfilling life were included, which partially align with the findings of this study (Law and Morrison 2014; Yamaguchi et al. 2020; Guerrero et al. 2024). These outcome domains seemed important for users of community mental health services regardless of their mental illness diagnosis. Some of these outcome domains also align with components of personal recovery (Van Weeghel et al. 2019), including ‘connectedness’ for social connectedness; ‘hope and optimism about the future’ for treatment adherence; ‘identity’ for self‐esteem; ‘meaning in life’ for life satisfaction, quality of life, or well‐being; ‘empowerment’ for empowerment or self‐determination; and ‘difficulties’ for adverse events or side effects. The identification of these outcome domains suggests that they are universally important in community mental health, across cultural differences and diagnostic categories.

### 4.2 Outcome Domains Unique to This Study

Unique outcome domains have also been identified. The outcome domains for which consensus was reached, help‐seeking and place of safety and belonging, had the highest agreement rates among all outcomes and were considered important across all stakeholder groups. Patients with mental disorders often experience feelings of loneliness and isolation when help‐seeking is not possible (da Rocha et al. 2018). While multiple meta‐analyses have shown that help‐seeking is associated with stigma (Clement et al. 2015; Schnyder et al. 2017), Japan has a stronger tendency toward stigma associated with mental illness than other countries (Ando et al. 2013). In addition, Japanese people with mental illness often face strong social norms and are sensitive to differences from others (Kanehara et al. 2022). These aspects of Japanese culture might be associated with challenges in help‐seeking and place of safety and belonging, potentially explaining the endorsement of these outcome domains.

In this study, we focused on service users with mental health conditions living in the community. However, the importance of ‘Help‐seeking’ and ‘Place of safety and belonging’ may extend beyond community settings. Individuals in institutional settings may experience fewer situations requiring help‐seeking, as they are in a more closely monitored environment that provides physical safety, which may contribute to a sense of security. However, caregivers and staff may not always fully understand service users' expectations and needs. Moreover, physical space does not necessarily equate to a safe and comfortable place. Therefore, while ‘Help‐seeking’ and ‘Place of safety and belonging’ may be more evident in community settings, we believe these elements are equally essential in institutional settings for individuals to lead fulfilling lives based on their choices and autonomy.

With regards to clinical outcomes, outcome domains that reached consensus in this study were broader, focusing on a comprehensive assessment of mental health, such as stress and general mental symptoms. A previous study (Tyler et al. 2020) identified specific symptoms of bipolar disorder, such as delusions, anxiety, depression, mood swings, and abnormal behaviour, as outcomes to be included in the COS. This difference might be due to limiting the number of items for consideration by not including all detailed symptoms as well as the fact that participants with a variety of mental illness diagnoses participated in the study. However, the result suggests that a comprehensive assessment of symptoms might be more important than a separate assessment of specific symptoms in the context of community mental health.

### 4.3 Stakeholder Group Characteristics and Degree of Agreement Among Groups

The multiple comparison results indicated significant differences in scores between two or more stakeholder groups for approximately 80% of outcome domains. These findings imply that stakeholder groups had differences in the perception of the importance of each outcome domain. Service users and caregivers considered medication adherence and treatment adherence significantly more important than service providers, government officials, and researchers. Despite the lack of consensus, service users and caregivers also rated clinical insight and knowledge of illness and services as significantly more important than the other groups. A previous study indicated that self‐care strategies, described with terms like ‘personal medicine’, enhance active participation in the recovery process (MacDonald‐Wilson et al. 2013). The service users and caregivers who participated in this study emphasised assessing treatment and clinical outcome domains as important for fulfilling community life, underscoring the high value they place on understanding and managing their illness.

The study also found that service users, service providers, and researchers valued satisfaction with life as well as autonomy and self‐determination more than caregivers and government officials, highlighting growing appreciation for support that aligns with personal recovery and user life choices (Thornicroft and Slade 2014). However, caregivers and government officials placed less importance on these domains, pointing to a need for greater awareness of personal recovery in Japan's home and residential settings.

In this study, the caregiver group rated the importance of all 18 outcome domains regarding caregivers significantly higher than the other stakeholder groups. Caregivers in Japan feel a strong sense of responsibility in caring for their patients (Hanzawa et al. 2010), but they also experience physical and psychological pain. In addition, they wish to have more free time for themselves (Mcauliffe et al. 2014). The present survey also showed that caregivers have high expectations of benefiting from support and services.

Overall, this study aligns with previous research showing diverse viewpoints on the significance of services among mental health service users, support professionals, and researchers (Collins 2019). On the other hand, this study consolidated responses across stakeholder groups to pinpoint key outcome areas. It revealed that despite differing priorities, domains like place of safety and belonging were universally deemed important (Table S3).

### 4.4 Strengths and Limitations

This study had several strengths. First, the number of participants and the response rate both exceeded their pre‐established targets, achieving a final response rate of 93.6% in three rounds of surveys with 297 participants. This rate is exceptionally high compared with previous studies, suggesting minimal bias in participant representation among stakeholder groups. Second, we incorporated the views of collaborators from diverse backgrounds during all stages of the research process. PPI is known to facilitate participant recruitment and the creation of research materials that are easily understood by participants (Crocker et al. 2018). The fact that this study attracted more participants than the target number and maintained a very high response rate might have been due to the implementation of PPI and collaboration with stakeholders, including those with lived experiences, during all stages.

This study has several limitations. First, the scope of the research articles reviewed during the development of the outcome domain list was restricted. The study included only systematic reviews and randomised controlled trials. While these types of studies are valuable for hypothesis testing, observational studies and practice reports might provide insights into more exploratory or novel outcomes. In addition, the study was conducted via an online survey, limiting participants to those with access to devices such as computers or smartphones and the capability to complete surveys online. This approach excluded individuals living in environments without such access. However, the COVID‐19 pandemic rendered face‐to‐face surveys impractical. Under these conditions, an online survey was considered an appropriate alternative. Furthermore, we aimed to reduce this bias by employing diverse recruitment methods, including outreach through various stakeholder organisations and publishing recruitment notices on the author's institutional website. We believe this strategy helped capture diverse stakeholder viewpoints as comprehensively as possible.

## 5 Conclusion

This study aimed to identify outcome domains considered important by multiple stakeholders in community mental health research in Japan. Through three rounds of surveys, 24 items reached consensus and were identified as important outcome domains. The items that reached consensus included elements aligned with comprehensive symptom assessment and aspects of personal recovery. Since community care serves patients with a wide variety of illnesses, outcome domains that are important across community care and not limited to specific illnesses would be useful for future research. This study also indicated that outcome domains considered important may be influenced by cultural factors. While it is important to seek a common set of outcomes internationally, it might also be important to examine essential outcome domains rooted in each culture. This study should be replicated in other countries in the future.

### 5.1 Relevance for Clinical Practice

The selection of appropriate outcome measures is frequently debated in routine clinical practice for monitoring patient prognosis. The 24 outcome domains identified through consensus in this study can serve as a valuable resource for clinical staff in community settings, facilitating more in‐depth discussions on outcome selection. Moreover, these findings can enhance clinical practice by enabling a more proper evaluation of services, thereby contributing to the improvement of community mental health services.

## Author Contributions

T.S. was the principal investigator responsible for the initial draft of this manuscript and the organisation and implementation of the study. C.F. secured the funding. All authors contributed to the data collection, data curation, or data analysis of at least one study. T.S., M.O., N.Y., and S.Y. conducted the analysis and compilation of the data. T.S., T.K., M.A., N.Y., and M.I. contributed to the data compilation, organisation, and preparation of materials. All authors reviewed and edited the manuscript and were responsible for the decision to submit for publication.

## Ethics Statement

The ethical considerations of the study, including the informed consent process and measures to protect patient privacy, were conducted in accordance with the Ethical Guidelines for Medical and Biological Research Involving Human Subjects issued by the Government of Japan.

## Conflicts of Interest

All authors are employed by or affiliated with the funding organisation.

## Supporting information


Data S1.

## Data Availability

The datasets generated and/or analysed during the current study are not publicly available because of the relevant Japanese policy for Ethical Guidelines for Medicine for a person of interest and the ethical committee approval for this study.

## References

[inm70049-bib-0001] Ando, S. , S. Yamaguchi , Y. Aoki , and G. Thornicroft . 2013. “Review of Mental‐Health‐Related Stigma in Japan.” Psychiatry and Clinical Neurosciences 67: 471–482. 24118217 10.1111/pcn.12086

[inm70049-bib-0002] Avella, J. R. 2016. “Delphi Panels: Research Design, Procedures, Advantages, and Challenges.” International Journal of Doctoral Studies 11: 305–321. 10.28945/3561.

[inm70049-bib-0003] Boardman, J. 2018. “Routine Outcome Measurement: Recovery, Quality of Life and Co‐Production.” British Journal of Psychiatry 212: 4–5. 10.1192/bjp.2017.5 29433606

[inm70049-bib-0004] Clement, S. , O. Schauman , T. Graham , et al. 2015. “What Is the Impact of Mental Health‐Related Stigma on Help‐Seeking? A Systematic Review of Quantitative and Qualitative Studies.” Psychological Medicine 45, no. 1: 11–27. 10.1017/S0033291714000129. 24569086

[inm70049-bib-0005] Collins, B. 2019. “Outcomes for Mental Health Services: What Really Matters?” The King's Fund.

[inm70049-bib-0006] COMET Initiative . 2011. “Core Outcome Measures in Effectiveness Trials.” https://www.comet‐initiative.org/.

[inm70049-bib-0007] Crocker, J. C. , I. RICCI‐Cabello , A. Parker , et al. 2018. “Impact of Patient and Public Involvement on Enrolment and Retention in Clinical Trials: Systematic Review and Meta‐Analysis.” British Medical Journal 363: k4738. 10.1136/bmj.k4738. 30487232 PMC6259046

[inm70049-bib-0008] da Rocha, B. M. , S. Rhodes , E. Vasilopoulou , and P. Hutton . 2018. “Loneliness in Psychosis: A Meta‐Analytical Review.” Schizophrenia Bulletin 44: 114–125. 10.1093/schbul/sbx036. 28369646 PMC5768045

[inm70049-bib-0009] Dalkey, N. C. 1969. The Delphi Method: An Experimental Study of Group Opinion. RAND Corporation.

[inm70049-bib-0010] Dodd, S. , S. L. Gorst , A. Young , S. W. Lucas , and P. R. Williamson . 2023. “Patient Participation Impacts Outcome Domain Selection in Core Outcome Sets for Research: An Updated Systematic Review.” Journal of Clinical Epidemiology 158: 127–133. 37054902 10.1016/j.jclinepi.2023.03.022

[inm70049-bib-0011] Fukasawa, M. , Y. Suzuki , S. Nakajima , K. Asano , T. Narisawa , and Y. Kim . 2015. “Systematic Consensus Building on Disaster Mental Health Services After the Great East Japan Earthquake by Phase.” Disaster Medicine and Public Health Preparedness 9: 359–366. 25905559 10.1017/dmp.2015.13

[inm70049-bib-0012] Gorst, S. L. , E. Gargon , M. Clarke , J. M. Blazeby , D. G. Altman , and P. R. Williamson . 2016. “Choosing Important Health Outcomes for Comparative Effectiveness Research: An Updated Review and User Survey.” PLoS One 11: e0146444. 26785121 10.1371/journal.pone.0146444 PMC4718543

[inm70049-bib-0013] Guerrero, E. , M. Barrios , H. M. Sampietro , A. Aza , J. GÓMEZ‐Benito , and G. Guilera . 2024. “Let's Talk About Recovery in Mental Health: An International Delphi Study of Experts by Experience.” Epidemiology and Psychiatric Sciences 33: e41. 10.1017/S2045796024000490. 39314142 PMC11464929

[inm70049-bib-0014] Hanzawa, S. , J.‐K. Bae , H. Tanaka , et al. 2010. “Caregiver Burden and Coping Strategies for Patients With Schizophrenia: Comparison Between Japan and Korea.” Psychiatry and Clinical Neurosciences 64: 377–386. 20546168 10.1111/j.1440-1819.2010.02104.x

[inm70049-bib-0015] Igarashi, M. , S. Yamaguchi , T. Kawaguchi , M. Ogawa , S. Sato , and C. Fujii . 2021. “Outcomes Frequently Specified in Cochrane Reviews of Community‐Based Psychosocial Interventions for Adults With Severe Mental Illness: A Systematic Search and Narrative Synthesis.” Neuropsychopharmacology Reports 41, no. 4: 459–463. 10.1002/npr2.12216. 34725970 PMC8698675

[inm70049-bib-0016] Jairath, N. , and J. Weinstein . 1994. “The Delphi Methodology (Part One): A Useful Administrative Approach.” Canadian Journal of Nursing Administration 7: 29–42. 7880844

[inm70049-bib-0017] Junger, S. , S. A. Payne , J. Brine , L. Radbruch , and S. G. Brearley . 2017. “Guidance on Conducting and REporting DElphi Studies (CREDES) in Palliative Care: Recommendations Based on a Methodological Systematic Review.” Palliative Medicine 31: 684–706. 28190381 10.1177/0269216317690685

[inm70049-bib-0018] Kanehara, A. , H. Koike , Y. Fujieda , et al. 2022. “Culture‐Dependent and Universal Constructs and Promoting Factors for the Process of Personal Recovery in Users of Mental Health Services: Qualitative Findings From Japan.” BMC Psychiatry 22: 105. 35144562 10.1186/s12888-022-03750-4 PMC8832737

[inm70049-bib-0019] Kelly, S. , L. Lafortune , N. Hart , K. Cowan , M. Fenton , and C. Brayne . 2015. “Dementia Priority Setting Partnership With the James Lind Alliance: Using Patient and Public Involvement and the Evidence Base to Inform the Research Agenda.” Age and Ageing 44, no. 6: 985–993. 10.1093/ageing/afv143. 26504119 PMC4621237

[inm70049-bib-0020] Law, H. , and A. P. Morrison . 2014. “Recovery in Psychosis: A Delphi Study With Experts by Experience.” Schizophrenia Bulletin 40: 1347–1355. 24727194 10.1093/schbul/sbu047 PMC4193718

[inm70049-bib-0021] Leamy, M. , V. Bird , C. L. Boutillier , J. Williams , and M. Slade . 2011. “Conceptual Framework for Personal Recovery in Mental Health: Systematic Review and Narrative Synthesis.” British Journal of Psychiatry 199, no. 6: 445–452. 10.1192/bjp.bp.110.083733. 22130746

[inm70049-bib-0022] MacDonald‐Wilson, K. L. , P. E. Deegan , S. L. Hutchison , N. Parrotta , and J. M. Schuster . 2013. “Integrating Personal Medicine Into Service Delivery: Empowering People in Recovery.” Psychiatric Rehabilitation Journal 36, no. 4: 258–263. 10.1037/prj0000027. 24320834

[inm70049-bib-0023] Mcauliffe, R. , L. O'connor , and D. Meagher . 2014. “Parents' Experience of Living With and Caring for an Adult Son or Daughter With Schizophrenia at Home in Ireland: A Qualitative Study.” Journal of Psychiatric and Mental Health Nursing 21: 145–153. 23593964 10.1111/jpm.12065

[inm70049-bib-0024] Mckenzie, E. , L. Matkin , L. SOUSA Fialho , et al. 2022. “Developing an International Standard Set of Patient‐Reported Outcome Measures for Psychotic Disorders.” Psychiatric Services 73, no. 3: 249–258. 10.1176/appi.ps.202000888. 34369809

[inm70049-bib-0025] Obbarius, A. , L. VAN Maasakkers , L. Baer , et al. 2017. “Standardization of Health Outcomes Assessment for Depression and Anxiety: Recommendations From the ICHOM Depression and Anxiety Working Group.” Quality of Life Research 26, no. 12: 3211–3225. 10.1007/s11136-017-1659-5. 28786017 PMC5681977

[inm70049-bib-0026] Retzer, A. , R. Sayers , V. Pinfold , et al. 2020. “Development of a Core Outcome Set for Use in Community‐Based Bipolar Trials—A Qualitative Study and Modified Delphi.” PLoS One 15, no. 10: e0240518. 10.1371/journal.pone.0240518. 33112874 PMC7592842

[inm70049-bib-0027] Sawada, U. , A. Matsunaga , A. Taneda , N. Sasaki , and S. Yamaguchi . 2024. “Perspectives of People With Schizophrenia on Clinical Outcome Scales and Patient‐Reported Outcome Measures: A Qualitative Study.” BMC Psychiatry 24, no. 1: 861. 10.1186/s12888-024-06292-z. 39614162 PMC11607935

[inm70049-bib-0028] Schnyder, N. , R. Panczak , N. Groth , and F. SCHULTZE‐Lutter . 2017. “Association Between Mental Health‐Related Stigma and Active Help‐Seeking: Systematic Review and Meta‐Analysis.” British Journal of Psychiatry 210, no. 4: 261–268. 10.1192/bjp.bp.116.189464. 28153928

[inm70049-bib-0029] Shiozawa, T. , S. Yamaguchi , M. Ogawa , et al. 2021. “Consensus Development of Priority Outcome Domains for Community Mental Health Cares by Multiple Stakeholders: Protocol for an Online Delphi Study in Japan.” Neuropsychopharmacology Reports 41: 1–8. 10.1002/npr2.12211 PMC8698667 34636183

[inm70049-bib-0030] Sinha, I. P. , R. L. Smyth , and P. R. Williamson . 2011. “Using the Delphi Technique to Determine Which Outcomes to Measure in Clinical Trials: Recommendations for the Future Based on a Systematic Review of Existing Studies.” PLoS Medicine 8: e1000393. 21283604 10.1371/journal.pmed.1000393 PMC3026691

[inm70049-bib-0031] Slade, M. , M. Amering , and L. Oades . 2008. “Recovery: An International Perspective.” Epidemiologia e Psichiatria Sociale 17: 128–137. 18589629 10.1017/s1121189x00002827

[inm70049-bib-0032] Smith, H. , A. Horobin , K. Fackrell , V. Colley , B. Thacker , and D. A. Hall . 2018. “Defining and Evaluating Novel Procedures for Involving Patients in Core Outcome Set Research: Creating a Meaningful Long List of Candidate Outcome Domains.” Research Involvement and Engagement 4: 8. 10.1186/s40900-018-0091-5. 29507772 PMC5833049

[inm70049-bib-0033] Thornicroft, G. , and M. Slade . 2014. “New Trends in Assessing the Outcomes of Mental Health Interventions.” World Psychiatry 13: 118–124. 24890055 10.1002/wps.20114 PMC4102275

[inm70049-bib-0034] Tyler, N. , N. Wright , A. Grundy , and J. Waring . 2020. “Developing a Core Outcome Set for Interventions to Improve Discharge From Mental Health Inpatient Services: A Survey, Delphi and Consensus Meeting With Key Stakeholder Groups.” BMJ Open 10: e034215. 10.1136/bmjopen-2019-034215 PMC7228512 32404388

[inm70049-bib-0035] Van Weeghel, J. , C. Zelst , D. Boertien , and I. Hasson‐Ohayon . 2019. “Conceptualizations, Assessments, and Implications of Personal Recovery in Mental Illness: A Scoping Review of Systematic Reviews and Meta‐Analyses.” Psychiatric Rehabilitation Journal 42, no. 2: 169–181. 10.1037/prj0000356. 30843721

[inm70049-bib-0036] Yamaguchi, S. , Y. Ojio , J. Koike , et al. 2024. “Associations Between Readmission and Patient‐Reported Measures in Acute Psychiatric Inpatients: A Multicenter Prospective Longitudinal Study.” Social Psychiatry and Psychiatric Epidemiology 60, no. 1: 79–93. 10.1007/s00127-024-02710-5. 39102067 PMC12987862

[inm70049-bib-0037] Yamaguchi, S. , T. Shiozawa , A. Matsunaga , et al. 2020. “Development and Psychometric Properties of a New Brief Scale for Subjective Personal Agency (SPA‐5) in People With Schizophrenia.” Epidemiology and Psychiatric Sciences 29: e111. 10.1017/S2045796020000256. 32272978 PMC7214545

[inm70049-bib-0038] Yamaguchi, S. , K. Usui , M. Iwanaga , et al. 2024. “10‐Year Outcome Trajectories of People With Mental Illness and Their Families Who Receive Services From Multidisciplinary Case Management and Outreach Teams: Protocol of a Multisite Longitudinal Study.” BMJ Open 14: e085532. 10.1136/bmjopen-2024-085532 PMC11367311 39298130

[inm70049-bib-0039] Yasuma, N. , T. Shiozawa , M. Ogawa , et al. 2022. “What Outcomes in Community Mental Health Research Are Important to Caregivers of People With Schizophrenia? An Exploratory Qualitative Analysis of an Online Survey.” Neuropsychopharmacology Reports 42: 526–531. 36217559 10.1002/npr2.12295 PMC9773637

[inm70049-bib-0040] Zendjidjian, X. Y. , and L. Boyer . 2014. “Challenges in Measuring Outcomes for Caregivers of People With Mental Health Problems.” Dialogues in Clinical Neuroscience 16: 159–169. 25152655 10.31887/DCNS.2014.16.2/xzendjidjian PMC4140510

